# Integrated Analysis of microRNAs and Transcription Factor Targets in Floral Transition of *Pleioblastus pygmaeus*

**DOI:** 10.3390/plants13213033

**Published:** 2024-10-30

**Authors:** Wenjing Yao, Peng Shen, Meng Yang, Qianyu Meng, Rui Zhou, Long Li, Shuyan Lin

**Affiliations:** 1 Co-Innovation Center for Sustainable Forestry in Southern China/Bamboo Research Institute, Nanjing Forestry University, 159 Longpan Road, Nanjing 210037, China; yaowenjing@njfu.edu.cn (W.Y.); shenpeng554@163.com (P.S.); emmy616@foxmail.com (M.Y.); mqy200816@163.com (Q.M.); kkismoon@163.com (R.Z.); lilong1949@126.com (L.L.); 2 State Key Laboratory of Tree Genetics and Breeding, Northeast Forestry University, Hexing Road, Harbin 150040, China

**Keywords:** *Pleioblastus pygmaeus*, floral transition, miRNAs, transcription factors, age pathway

## Abstract

Bamboo plants have erratic flowering habits with a long vegetative growth and an uncertain flowering cycle. The process of floral transition has always been one of the hot and intriguing topics in bamboo developmental biology. As master modulators of gene expression at the post-transcriptional level, miRNAs play a crucial role in regulating reproductive growth, especially in floral transition of flowering plants. *Pleioblastus pygmaeus* is a kind of excellent ground cover ornamental bamboo species. In this study, we performed miRNA expression profiling of the shoot buds and flower buds from the bamboo species, to investigate flowering-related miRNAs in bamboo plants. A total of 179 mature miRNAs were identified from *P. pygmaeus*, including 120 known miRNAs and 59 novel miRNAs, of which 96 (61 known miRNAs and 35 novel miRNAs) were differentially expressed in the shoots at different growth stages. Based on target gene (TG) prediction, a total of 2099 transcription factors (TFs) were annotated to be TGs of the 96 differentially expressed miRNAs (DEMs), corresponding to 839 recordings of DEM-TF pairs. In addition, we identified 23 known DEMs involved in flowering and six known miRNAs related to floral organ development based on previous reports. Among these, there were 11 significantly differentially expressed miRNAs, with 124 TF targets corresponding to 132 DEM-TF pairs in *P. pygmaeus*. In particular, we focused on the identification of *miR156a*-SPL (SQUAMOSA Promoter-Binding protein-Like) modules in the age pathway, which are well-known to regulate the vegetative-to-reproductive phase transition in flowering plants. A total of 36 TF targets of *miR156a* were identified, among which there were 11 SPLs. The Dual-Luciferase transient expression assay indicated *miR156a* mediated the repression of the PpSPL targets in *P. pygmaeus*. The integrated analysis of miRNAs and TGs at genome scale in this study provides insight into the essential roles of individual miRNAs in modulating flowering transition through regulating TF targets in bamboo plants.

## 1. Introduction

Floral transition (vegetative-to-reproductive transition phase) is a critical and distinctive stage in the life cycle of plants, which ensures their reproductive success [1]. Floral transition is synergistically induced by diverse endogenous cues, such as phytohormones and carbohydrate status, and dynamic external signals, such as light and low temperature, which are regulated by multiple regulatory pathways involving these internal and external signals [2,3]. A common set of floral pathways contain many general gene regulators, which target the genes with a specific flowering function [4,5]. Known as master modulators of gene expression at the post-transcriptional level, microRNAs (miRNAs) are non-coding RNAs with 20–24 nucleotides, which can negatively regulate multiple mRNA targets by either mRNA degradation or translational suppression [6]. Many miRNAs are evolutionarily conserved in plants, participating in numerous essential biological processes including vegetative tissue development, reproductive process, and abiotic stress response [7]. In particular, increasing reports signify the paramount importance of miRNAs in juvenile-to-adult transition, floral organ formation, and flowering time determination [8,9,10].

Notably, the majority of miRNA targets are transcription factors (TFs), which also play crucial roles in plant growth and development [11]. In general, TFs can up-regulate or down-regulate dozens of target genes through activating or repressing their transcription in gene networks [12]. miRNAs and TFs demonstrate a great impact on the plant genetic system at the post-transcriptional level and transcriptional level, respectively [13]. It is worthy to explore the dynamic expression pattern of miRNA-TF modules in the multiple biological processes. To date, the evolutionarily conserved roles of miRNA-TF interactions in floral transition have been extensively confirmed in many plant species [14,15,16,17,18,19,20,21]. The most typical pairs are *miR156* and SPL (SQUAMOSA Promoter-Binding protein-Like) TF targets in the age pathway [14,15]. In general, *miR156* functions to promote juvenile development, while SPL targets function to accelerate adult development [14]. Meanwhile, *miR156* remains at a high expression level in young seedlings and consequently declines before floral induction, which represses the expression of SPL targets during the juvenile phase [15]. Another critical miRNA in the age pathway is *miR172*, which is regulated by several SPLs and represses a class of *APETALA2* (*AP2*)-like TFs to promote the vegetative phase change, exhibiting a complementary function to *miR156* [16]. The *miR156*-SPL-*miR172* module is a decisive factor in the vegetative-to-reproductive phase transition in plants [17,18]. In addition, an adequate amount of evidence highlights the critical roles of other miRNAs such as *miR159*, *miR164–167*, *miR169*, *miR319*, *miR390*, *miR396,* etc., and their TF targets in promoting or repressing flowering in plants [7,9,10]. For example, over-expression of *miR159* causes a decreased expression of LEAFY (*LFY*) and Myeloblastosis 33 (*MYB33*) in transgenic *Sinningia speciosa*, resulting in late flowering [19]. *miR169* regulates stress-induced early flowering by repressing the expression of NUCLEAR FACTOR Y Subunit A 2 (*NF-YA2*) TF [20]. *miR390* prolongs the juvenile phase by inhibiting the Auxin Response Factor 3 (*ARF3*) TF in *Arabidopsis thaliana* [21].

As one of the renewable forestry resources, bamboo plants are widely used for ornamental, architectural, and agricultural uses, in the water conservancy, papermaking, environmental protection, handicraft, and furniture industries, in culture and art, and in many other areas [22]. However, bamboo plants display special reproductive characteristics with a prolonged vegetative phase, an unpredictable flowering cycle, and rare seed production [23,24]. Beyond this, the entire part of the plant that stands above ground of many bamboo species die after flowering, leading to a highly low likelihood of obtaining their reproductive organs [23,24]. In particular, the large-scale death after blooming limits the sustainable development of bamboo forests [23]. Furthermore, most of the bamboo species lack an efficient genetic transformation system, which also greatly hinders the molecular biological research and genetic breeding process of bamboo species [25]. Therefore, despite the significant roles of bamboo plants in many aspects, the molecular mechanism research on their flowering lags behind other common flowering plants, particularly the model plants such as Arabidopsis and rice.

*Pleioblastus pygmaeus* belongs to Pleioblastus Nakai of Bambusoideae, Poaceae, which is a kind of excellent ground cover ornamental bamboo species. It is widely used in landscaping and has high economic and ornamental values [26]. In recent years, *P. pygmaeus* has been flowering continuously in the bamboo garden of Nanjing Forestry University, which provides a great opportunity to explore its flowering process comprehensively. In our previous studies, we observed the morphological structure of flower organs and investigated flower bud differentiation and whole reproductive stages of *P. pygmaeus* systematically [24,26]. Moreover, we identified a total of 129 flowering-related genes by transcriptome comparisons of the shoot buds at the different growth stages in flowering *P. pygmaeus* [26,27]. The above results provide the theoretical basis for the investigation of the dynamics of miRNAs and their biological functions during these growth and flowering stages in *P. pygmaeus*. In this study, small RNA (sRNA) expression profiling was performed on the shoot buds of *P. pygmaeus* at different growth stages by high-throughput sequencing. A total of 96 differentially expressed miRNAs (DEMs) were identified, including 23 flowering-related miRNAs and 6 known miRNAs related to floral organ development. Among the 23 flowering-related miRNAs, 11 were identified to be closely related to floral transition. In particular, we focused on the identification of miR156a-SPL TF modules in the age pathway, which are well-known in regulating the vegetative-to-reproductive phase transition in plants. A total of 36 TF targets of *miR156a* were identified, among which there were 11 SPLs. Here we demonstrated that the *miR156a*-mediated repression of *PpSPL* targets occurs in *P. pygmaeus* by the Dual-Luciferase transient expression assay. In this study, the genome-wide profiling of miRNAs and the exploration of their TF targets across the vegetative-to-reproductive transition phase of *P. pygmaeus* shed light on their functions in bamboo flowering.

## 2. Materials and Methods

### 2.1. Plant Materials

In recent years, *P. pygmaeus* plants have flowered under a natural environment during March to April in the bamboo garden of Nanjing Forestry University (32°4′44″ N, 118°48′17″ E) in Jiangsu Province, China. The shoot buds (F1) and flower buds (F2) from 30 clusters of flowering *P. pygmaeus* and the shoot buds (N1) and leaf buds (N2) from 30 clusters of non-flowering plants were harvested with six respective biological replicates (Figure S1). The four tissues with three respective biological replicates were sent with dry ice to the Novogene company (https://cn.novogene.com/ (accessed on 29 November 2023)) for sRNA sequencing. And the remaining tissues were frozen in liquid nitrogen and stored in a –80 °C refrigerator for RT-qPCR.

### 2.2. RNA Preparation and sRNA Library Generation

Total RNA was obtained using the Trizol method (Invitrogen, Waltham, MA, USA). RNA purity was checked by a NanoPhotometer^®^ spectrophotometer (IMPLEN, Westlake Village, CA, USA). RNA quantity was measured by the Qubit^®^ RNA Assay Kit in the Qubit^®^ 2.0 Flurometer (Life Technologies, Carlsbad, CA, USA). RNA integrity was determined by the RNA Nano 6000 Assay Kit in the Agilent Bioanalyzer 2100 system (Agilent Technologies, Santa Clara, CA, USA).

Total RNA (3 μg per sample) was prepared for sRNA library generation using the NEBNext^®^ Multiplex Small RNA Library Prep Set for Illumina^®^ (NEB, Ipswich, MA, USA). Library quality was assessed on the Agilent Bioanalyzer 2100 system using DNA High Sensitivity Chips. A total of 12 small RNA libraries were successfully constructed with three biological replicates of each tissue, which were then sequenced on an Illumina Hiseq 2500/2000 platform (Novogene company, Beijing, China).

### 2.3. Data Control and miRNA Annotation

Raw sequence data were obtained in Fastq format. And clean sequence data were generated by removing the low-quality reads containing poly-N and poly-A or -T or -G or -C, with 5′ adapter contaminants, without 3′ adapters or the insert tag, and shorter than 18 nt (not including adapters), from the raw sequence reads through the processing of custom Perl and Python scripts. By mapping the sRNA tags with lengths of 18–30 nt to the Repeat Masker (http://www.girinst.org/ (accessed on 18 December 2023)), GenBank (https://www.ncbi.nlm.nih.gov/ (accessed on 18 December 2023)) and Rfam (http://rfam.xfam.org/ (accessed on 18 December 2023)) databases using BOWTIE (http://bowtie-bio.sourceforge.net/ (accessed on 18 December 2023)), the tags originating from protein-coding genes, repeat sequences, rRNA, tRNA, snRNA, and snoRNA were removed from the clean data to obtain potential miRNA tags. As sRNA tags were mapped to more than one category, we made the following priority rule to ensure each unique sRNA mapped to only one annotation: known miRNA > rRNA > tRNA > snRNA > snoRNA >repeat gene > NAT-siRNA > gene > novel miRNA > ta-siRNA. The total rRNA proportion was used as the sample quality indicator, with less than 60% as high quality.

The remaining miRNA tags aligned to the miRNA sequences of miRBase (http://www.mirbase.org (accessed on 26 December 2023)) by BOWTIE [28] were used to identify known miRNAs. In general, the miRNA tags can be mapped to the reference genome without mismatch to analyze their expression and distribution. As the reference genome information of *P. pygmaeus* has not been published and recorded to date, we mapped them on the reference sequence of *Oryza sativa* L. in this study. The srna-tools-cli (http://srna-workbench.cmp.uea.ac.uk (accessed on 26 December 2023)) and mirdeep2 [29] tools were used to obtain their secondary structures and screen potential miRNAs, respectively. The identified known miRNAs were uploaded to miFam.dat (https://www.mirbase.org/ (accessed on 26 December 2023)) to confirm their miRNA families. In addition, the secondary structure of miRNA precursors of unannotated sRNA tags can be used to identify novel miRNAs. The miREvo [30] and mirdeep2 tools were integrated to predict novel miRNAs through exploring their hairpin structures, dicer cleavage sites, and minimum free energy. The identified novel miRNA precursors were submitted to Rfam (https://rfam.org/ (accessed on 26 December 2023)) to find their Rfam families.

### 2.4. DEM Identification

Custom scripts were used to obtain miRNA counts as well as base biases of all the identified miRNAs. The expression level of individual miRNA was normalized by TPM (transcript per million) through the following criteria: normalized miRNA expression = (mapped read count/total reads) × 1,000,000. The identification of significantly differentially expressed miRNAs (DEMs) was performed using the DESeqR package with padj < 0.05 [31,32]. And the identified DEMs were clustered based on TPM by Heatmapper (http://www.heatmapper.ca/expression/ (accessed on 30 December 2023)) with default parameters.

### 2.5. RT-qPCR Validation

Total RNA was extracted by the miRcute Plant miRNAIsolation Kit (Tiandz, Beijing, China). The quantity and quality of RNA were determined and assessed by Nanodrop 2000c and Agilent 2100, respectively. The 1st strand cDNA was obtained using miRNA 1st Strand cDNA Synthesis Kit (by stemoop) (Vazyme, Nanjing, China). U6 snRNA was used as the internal reference gene [33] and miRNA specific stem-loop primers were designed according to the description of Chen et al. 2005 [34] (Table S1). RT-qPCR was performed with the miRNA Universal SYBRgPCR Master Mix (Vazyme, Nanjing, China) in an ABI StepOne Plus Real-Time PCR system (ABI, Alexandria, VA, USA). Three independent biological repeats were prepared for each sample. The relative expression levels of miRNAs was calculated using the 2^−ΔΔCT^ method. Standard errors and standard deviations were calculated by ANOVA, with *p*-values ≤ 0.05 as the statistically significant level.

### 2.6. TG Prediction and DEM-TG Interaction Validation

Target gene (TG) prediction of all identified DEMs was performed by the psRobot tar in psRobot [35]. The potential TGs of conserved flowering-related DEMs were identified by psRNATarget (https://www.zhaolab.org/psRNATarget/analysis (accessed on 30 December 2023)) and TAPIR (http://bioinformatics.psb.ugent.be/webtools/tapir/ (accessed on 30 December 2023)). The link graph of *miRNA156a* and its TF targets was drawn by R with the Spearman function.

Total RNA was isolated from the flower buds of *P. pygmaeus* using the RNAprep Pure Plant Plus kit (TIANGEN, Beijing, China). And 1 μg total RNA was reverse transcribed into cDNA using the TIANScript II RT Kit (TIANGEN, Beijing, China). *PpSPL* transcript sequences were obtained by using Phanta Max Super-Fidelity DNA Polymerase (Vazyme, Nanjing, China) with specific primer pairs (Table S1). Then the *PpSPLs* sequences with predicted target sites were inserted into pGreenII 0800-miRNA vectors by the TA/Blunt-Zero Cloning Kit (Vazyme, Nanjing, China). The precursor sequence of *miR156a* (ped-*miR156a*) was inserted into the pGreenII 62-SK vector, driven by the 35S promoter. The recombinant vectors including pGreenII-0800::*PpSPLs* and pGreenII::ped-*miR156a* were transformed into GV3101 (pSoup). The positive Agrobacteria strains with OD_600_ of 0.5 were co-transformed into the abaxial surface of the *Nicotiana benthamiana* leaves. After 48 h of incubation in the dark, the fluorescent signals in the leaves were detected under a fluorescent microscope (Olympus, Tokyo, Japan).

### 2.7. GO and KEGG Enrichment Analysis

Gene Ontology (GO) enrichment analysis and Kyoto Encyclopedia of Genes and Genomes (KEGG) pathway annotation were performed on the DEM-TG modules to explore their regulatory roles in flowering. The GOseq-based Wallenius non-central hyper-geometric distribution was implemented for the GO enrichment analysis of TGs [36]. The statistical enrichment of TGs in KEGG pathways was tested by the KOBAS v2.0 [37]. GO enrichment and pathway were conducted with a threshold of corrected *p*-value <0.05 in the GO and KEGG databases, respectively.

## 3. Results

### 3.1. Identification and Classification of sRNAs in P. pygmaeus

**Figure 1:**
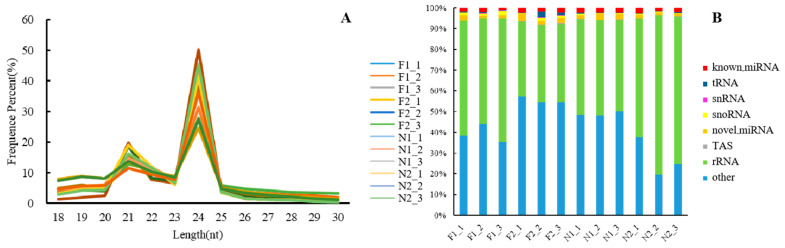
Identification and classification of sRNAs in *P. pygmaeus*. (**A**) The distribution of small RNA with 18–30 nt length in each sample. (**B**) The classification of annotated sRNAs in *P. pygmaeus.* F1_1–3, the shoot buds of flowering *P. pygmaeus* with three biological repeats; F2_1–3, the flower buds of flowering *P. pygmaeus* with three biological repeats; N1_1–3, the shoot buds of non-flowering *P. pygmaeus* with three biological repeats; N2_1–3, the leaf buds of non-flowering *P. pygmaeus* with three biological repeats.

In this study, we successfully constructed 12 sRNA libraries of the shoot buds (F1) and flower buds (F2) from flowering *P. pygmaeus* and the shoot buds (N1) and leaf buds (N2) from non-flowering plants with three respective biological repeats, which generated 12,041,474~15,237,465 raw reads and 11,903,935~14,787,443 clean reads in total. By screening the clean reads, we obtained 9,062,656~11,767,711 total reads of sRNA in 18~30 nt, including 2,013,978~3,515,032 unique sRNAs. Among them, 24 nt sRNAs accounted for the greatest proportion, about 31.53% of total sRNAs (Figure 1A). A total of 6,225,589, 3,890,829, 5,106,291, and 5,974,611 sRNAs can be mapped to the reference databases, accounting for 60.37%, 40.18%, 49.39%, and 63.32% of the total sRNA reads in each tissue.

The sRNAs mapped to the databases were classified and annotated, resulting in different kinds of sRNAs (Figure 1B). Among them, rRNAs accounted for the highest proportion, with 55.47%, 37.07%, 45.41%, and 69.24% of the total reads in each tissue. It was followed by the unannotated sRNAs, accounting for 40.92% of the total reads. A total of 1,235,599 and 1,276,391 sRNAs were identified to be known miRNAs and novel miRNAs, accounting for 1.94% and 2.01% of the total reads, respectively. The proportions of other types of sRNAs, such as tRNAs, snRNAs, and snoRNAs, were 0.52%, 0.08%, and 0.91%, respectively.

### 3.2. Identification and Classification of miRNAs in P. pygmaeus

**Figure 2:**
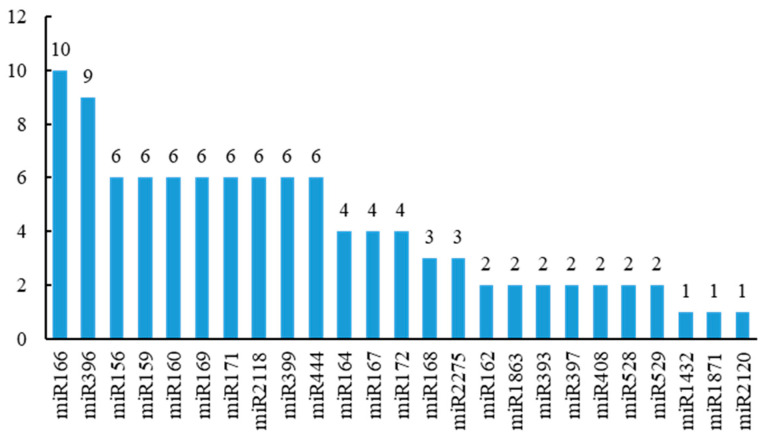
The classification of miRNA families in *P. pygmaeus*.

A total of 120 known miRNAs were identified from *P. pygmaeus*, with 138 precursors. The length of known miRNAs was 20–24 nt, and 67.5% of them displayed a length of 21 nt. The mature sequences of these miRNAs were completely identical to those of the miRNAs from rice, belonging to 43 highly conserved miRNA families in plants, such as *miR156*, *miR159*, *miR160*, *miR169*, *miR171*, *miR172*, *miR396,* etc. (Table S2). There were different numbers of family members in various miRNA families, ranging from 1 to 10. The *miR166* and *miR396* families have 10 and 9 members, respectively. It was followed by the *miR156*, *miR159*, *miR160*, *miR169*, *miR171*, *miR2118*, *miR399*, and *miR444* families, with six members each. There were four members in the *miR164*, *miR167,* and *miR172* families, each. The remaining known miRNA families had three or less members (Figure 2).

A total of 59 novel miRNAs were identified in *P. pygmaeus* based on the unique hairpin structure of 68 miRNA precursors. The length of the mature sequence of novel miRNAs ranged from 18 to 25 nt, with 24 nt being the most abundant, accounting for 50.85% of all novel miRNAs (Table S3). The novel miRNAs were not classified into any miRNA family, indicating there are still many unknown miRNAs whose functions need to be explored in *P. pygmaeus*.

### 3.3. Identification of Differentially Expressed miRNAs in P. pygmaeus

**Figure 3:**
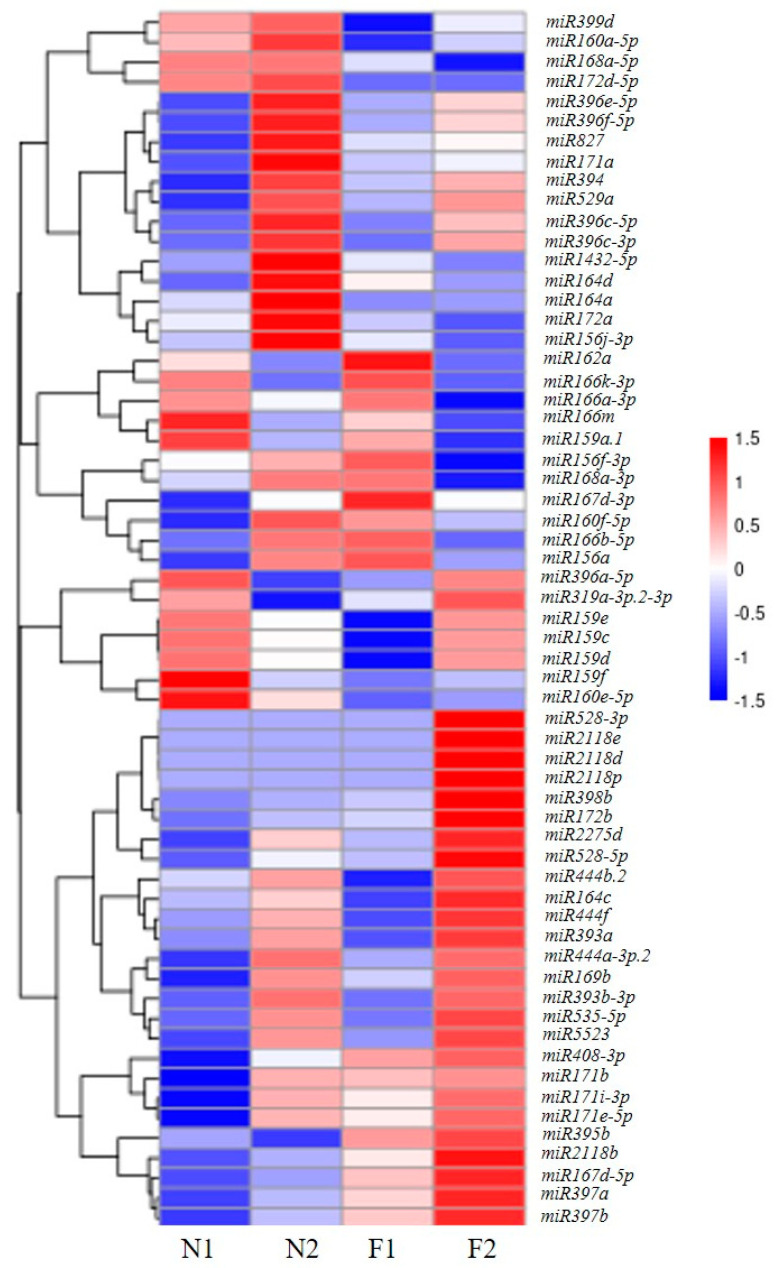
Cluster heatmap of 61 differentially expressed known miRNAs. F1, the shoot buds of flowering *P. pygmaeus*; F2, the flower buds of flowering *P. pygmaeus*; N1, the shoot buds of non-flowering *P. pygmaeus*; N2, the leaf buds of non-flowering *P. pygmaeus*.

A total of 96 differentially expressed miRNAs (DEMs) were identified in the shoots at different stages, including 61 known miRNAs and 35 novel miRNAs. As shown in Figure 3, we conducted a clustering analysis of the 61 known DEMs in the four tissues of *P. pygmaeus*. There were 19 DEMs between F1 and N1, of which 10 were up-regulated and 9 were down-regulated. A total of 35 DEMs were identified in the comparison pairs of F2 and N2. And 16 of them were up-regulated and the remaining 19 were down-regulated. Comparing the two tissues of flowering *P. pygmaeus* (F1 vs. F2), 21 DEMs were profiled, including 7/14 up-/down-regulated miRNAs. There were 25 DEMs between the two tissues of non-flowering *P. pygmaeus* (N1 vs. N2), including 10/25 up-/down-regulated known miRNAs (Supplemental Excel S1).

### 3.4. Go and KEGG Analysis of Candidate TGs of DEMs in P. pygmaeus

In this study, there were 3332, 5650, 3611, and 7050 candidate TGs of DEMs predicted in the F1 vs. N1, F2 vs. N2, F1 vs. F2, and N1 vs. N2 comparison pairs, respectively. We conducted GO classification and KEGG enrichment analysis of the candidate TGs corresponding to the DEMs in each pair. The GO analysis showed the TGs corresponding to DEMs in F1 vs. N1 and F2 vs. N2 were mainly enriched in molecular functions such as binding and nucleoside-triphosphatase activity, while the TGs of DEMs in F1 vs. F2 and N1 vs. N2 were mainly enriched in cellular components such as in the cell and intracellular parts (Figure S2). The KEGG analysis indicated that all the identified TGs were mainly enriched in multiple synthesis and metabolic pathways, such as cysteine and methionine metabolism, monocycline biosynthesis, arginine biosynthesis, glyoxylate and dicarboxylic acid metabolism, etc. (Figure S3). Among all the identified TGs, a total of 2099 transcription factors (TFs) were annotated, of which 132, 96, 93, 44, 166, 117, 19, 56, 87, and 13 belong to the known TF families including AP2/ERF (APETALA2/Ethylene Response Elements), bHLH (basic Helix-Loop-Helix), bZIP (basic leucine Zippers), MADS (MCM1 AGAMOUS DEFICIENS SRF), MYB, NAC (NAM, ATAF1/2, CUC1/2), SPL, TCP (Teosintebranched l/Cycloidea/Proliferating cell factor), WRKY (WRKYGOK), and GRF (Growth-Regulating Factor), respectively (Supplemental Excel S2). In addition, the TG prediction analysis showed that there were 839 recordings of miRNA-TF pairs (Supplemental Excel S2).

### 3.5. Identification of DEMs and TGs in Flowering and Flower Organ Development in P. pygmaeus

Increasing evidence confirmed the conserved functions of miRNAs in flowering and flower organ development. Based on the current research reports, we identified 23 conserved DEMs involved in plant flowering, belonging to 11 miRNA families (Table S1). Among them, 11 out of the 23 miRNAs were significantly differentially expressed between the shoot buds and flower buds, including *miR156a* (delayed flowering), *miR159f* (delayed flowering), *miR168a-5p* (reduced flowering time), *miR169b* (early flowering), *miR171a* (late flowering), *miR172b* (early flowering), *miR393b-3p* (early flowering), *miR394* (delayed flowering), *miR397a* (delayed flowering), *miR399d* (early flowering), and *miR528-3p* (early flowering) (Table S4). And the 11 miRNAs were predicted to have 124 TF targets, corresponding to 132 DEM-TF pairs. In particular, the two known miRNAs in the age pathway [14,15], *miR156a* and *miR172b*, target 36 and 24 TFs, including 11 SPL and two AP2-like genes, respectively (Supplemental Excel S2). Many miRNA-targeting genes have been demonstrated to be involved in flower development (Table S4). Meanwhile, we identified six known miRNAs related to floral organ development, including *miR164c* (sepal/sepal boundary growth), *miR167d-5p* (anther dehiscence and ovule development), *miR2118* (*miR2118d*, *miR2118e*, and *miR2118p*, photoperiodic sterility), and *miR396a-5p* (floral organ separation) (Table S5).

### 3.6. Validation of Two Known miRNAs in Age Pathway by qRT-PCR

**Figure 4:**
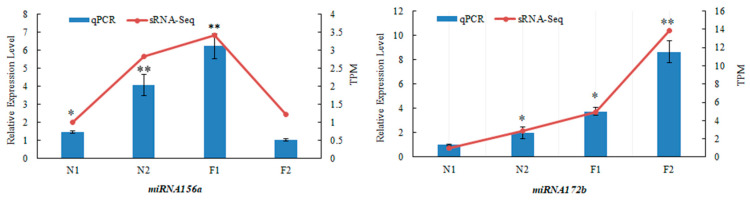
Expression pattern of *miRNA156a* and *miRNA172b* in *Pleioblastus pygmaeus.* F1, the shoot buds of flowering *P. pygmaeus*; F2, the flower buds of flowering *P. pygmaeus*; N1, the shoot buds of non-flowering *P. pygmaeus*; N2, the leaf buds of non-flowering *P. pygmaeus.* * means *p* < 0.05, ** means *p* < 0.01.

We conducted qRT-PCR to validate the expression pattern of the two known miRNAs participating in the age pathway in *Pleioblastus pygmaeus*. As shown in Figure 4, the relative expression level of *miR156a* was significantly higher in the shoot buds than in the flower buds, while it displayed an opposite trend for *miRNA172b*. In the non-flowering plants, the expression of both *miR156a* and *miRNA172b* was higher in the leaf buds than in the shoot buds. Overall, the expression trends of the two miRNAs quantified by qRT-PCR were generally consistent with the RNA-Seq analysis (Figure 4).

### 3.7. Identification of miR156a-PpSPLs Modules in P. pygmaeus

**Figure 5:**
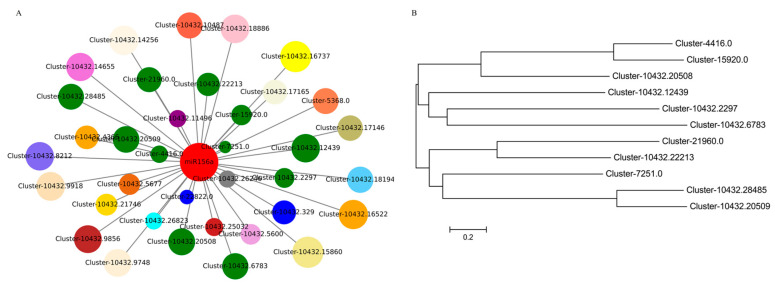
Link graph of *miRNA156a* and TF targets and phylogenetic analysis of 11 *miRNA156a*-targeting PpSPLs. (**A**) Link graph of *miRNA156a* and TF targets. Different colors represent different family genes. Red icon represents *miRNA156a* and green icons represent 11 putative PpSPLs. The circle size means the mRNA abundance of genes, the line distance means the regulatory role of *miRNA156a* in down-regulating TF targets. (**B**) Phylogenetic analysis of the 11 putative *miRNA156a*-targeting PpSPLs.

**Figure 6:**
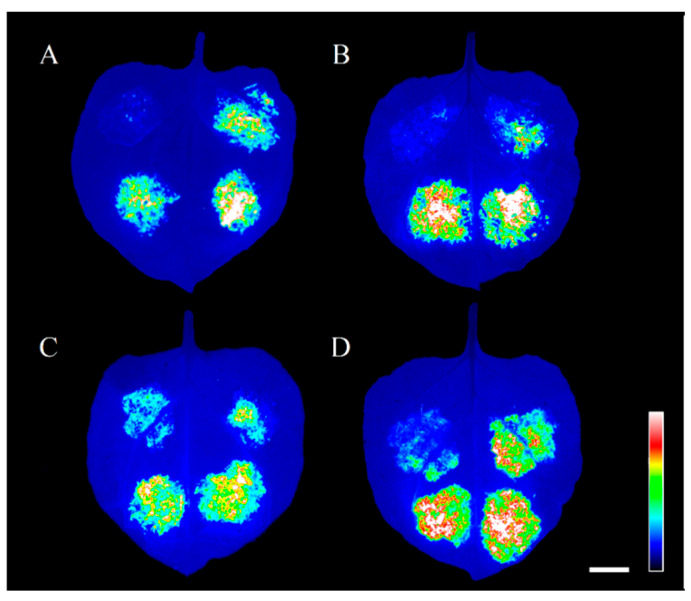
The correlation of *miR156a* with four putative *PpSPL* targets using the Dual-Luciferase transient expression assay. (**A**–**D**) Fluorescence scanning of tobacco leaves co-transforming *miR156a* with *PpSPL12* (Cluster-10432.20508), *PpSPL13* (Cluster-10432.12439), *PpSPL14* (Cluster-10432.2297), and *PpSPL16* (Cluster-10432.22213), respectively. Note: the same leaf injected with pGreenII-0800-PpSPL+ 35s-miR156-NOS are shown in the top left part, pGreenII-0800-PpSPL+ 35s-NOS in the top right part, pGreenII-0800+35s-miR156-NOS in the bottom left part, and pGreenII-0800+35s-NOS in the bottom right part. Bar = 1 cm.

In our previous study, we identified a total of 28 SPLs from *P. pygmaeus* by RNA-Seq, and 20 of them were identified to be involved in the age pathway [26]. TG prediction using psRNATarget software indicated *miR156a* had a cleavage effect on 13 out of the 20 SPL members [27]. In this study, we identified a total of 36 TF targets of *miR156a*, among which there were 11 belonging to SPL TFs (Figure 5A). Base alignment indicated there were corresponding binding sites between *miR156a* and the 11 putative *PpSPLs* (Supplemental Excel S2). Phylogenetic analysis indicated the expression of the putative *PpSPLs* with close relationships was repressed by *miR156a* similarly (Figure 5). For example, the mRNA abundance of Cluster-10432.28485 and Cluster-10432.20509, whose homologous relationship was close, was analogous in the shoot buds. It displayed the same phenomenon for Cluster-10432.22213 and Cluster-21960 (Figure 5). We chose four out of them (*PpSPL12*, *PpSPL13*, *PpSPL14*, and *PpSPL16*) to validate their interaction with *miR156a* by using the Dual-Luciferase transient expression assay in tobacco. As shown in Figure 6, the co-transformation of pGreenII-0800::PpSPLs and pGreenII::ped-miR156a resulted in lower fluorescence intensity than those of the controls, indicating *miR156a* mediated the repression of the four PpSPL targets in *P. pygmaeus*.

## 4. Discussion

Floral transition has always been one of the intriguing topics in bamboo developmental biology. Since the 1990s, extensive efforts have been made to unravel the mystery of bamboo flowering based on the increasing flowering events of several bamboo species [25]. Previous studies have mainly focused on the developmental biology research of bamboo plants, such as somatic embryogenesis, gametogenesis, flowering bud differentiation, floral organ structure, fertilization, etc. [26]. With increasing bamboo species being sequenced, it opens up a new path to explore the flowering mechanism of bamboo plants by high-throughput sequencing [38,39]. For instance, genomic analysis reveals genetic clues to the long vegetative growth phase of woody bamboos, including the loss/malfunction of *SOC1* (Suppressor of Overexpression of Constans 1)-like genes, positive selection of *OsPRR95* (pseudo-response regulator)-like genes, and copy number variation of GA pathway enzymes [39].

Increasing scientific evidence confirms various miRNAs play a vital role in regulating flowering and flower organ development at the post-transcriptional level in flowering plants [8,9,10]. In the recent decade, high-throughput sRNA sequencing empowers researchers to yield significant advancements in the identification of flowering-related miRNAs in *Phyllostachys edulis* (moso bamboo). For example, a total of 409 conserved miRNAs and 492 novel miRNAs were profiled to be differentially expressed at different flowering developmental stages of moso bamboo by using Illumina technology [33]. In the study of Ge et al. 2017 [40], six miRNAs were confirmed to be significant regulators in floral transition and flower development in moso bamboo. However, few studies have been reported on the systematic investigation of miRNAs in other bamboo species to date. In our study, we conducted a comprehensive miRNA profile in *Pleioblastus pygmaeus* and identified 96 differentially expressed miRNAs by sRNA sequencing. Among these, there were 11 pivotal miRNAs significantly differentially expressed between floral tissues and vegetative tissues in flowering *P. pygmaeus*. These findings provide the first broad survey of the microRNAomes of *P. pygmaeus*, which contributes to the identification of flowering-related miRNAs in bamboo plants.

In general, miRNAs negatively regulate their targeted genes (TGs) through transcript cleavage or translation repression [6,11]. Therefore, the biological functions of miRNAs can also be speculated by exploring their TGs [31]. Interestingly, many TGs of miRNAs encode transcription factors (TFs) in plants, for example, *miR156* is well-known to target SPLs, *miR159* targets MYBs, and *miR172* targets AP2s, indicating miRNAs function by regulating TF targets in plants [11,13]. There have been several studies addressing the roles of miRNAs and their TGs in the various developmental processes of bamboo plants, such as shoot development, lignification, and culm color [41,42,43]. Nonetheless, only a limited number of reports delineate the functions of miRNAs and their TF targets in bamboo flowering. For example, a total of 165 miRNAs were screened from the developing flowers of *Dendrocalamus latiflorus*, and fifteen out of them were specific to inflorescence development. The identified miRNAs targeted as many as 130 floral unigene candidates, and most of them encoded TFs [44]. It was revealed by the use of transgenic Arabidopsis, in which the overexpression of *miR159* from moso bamboo affected anther dehiscence and reduced the expression of the TF target, *AtMYB33* [45]. In this study, a total of 2099 TFs were predicted to be the TGs of the conserved miRNAs in *P. pygmaeus*, of which 124 TFs were identified to be the putative TGs of the 11 pivotal miRNAs that may be closely related to flowering. The data in this study underscore the substantial role of miRNAs and their TF targets in *P. pygmaeus* flowering.

The *miR156* and *miR172* genes are master regulators of the ageing pathway, acting sequentially to control the onset of reproductive competency in plants [14,15]. In general, the two known miRNAs exhibit a complementary expression pattern during the vegetative-to-reproductive transition phase in plants [17,46]. *miR156* is known to prolong the vegetative phase by repressing the expression of SPLs, while *miR172* promotes the phase transition by up-regulating AP2-like genes [16,17]. In this study, the relative expression level of *miR156a* was significantly higher in the shoot buds than that in the flower buds in flowering *P. pygmaeus*, which is similar to the significant down-regulation of *miR156* during the transition from the vegetative to flowering stages in moso bamboo [33], while it displayed the temporally opposite expression trend for *miR172b*, which was more highly expressed in the flower buds than in the shoot buds in *P. pygmaeus*, which is also in agreement with the expression pattern of *miR172* in moso bamboo [33]. In addition, we identified a total of 11 *miR156a*-targeting SPLs and two *miR172b*-targeting AP2-like TFs in *P. pygmaeus*. Gene annotation indicated the identified TFs were also associated with flowering or flower organ development in plants. Moreover, we confirmed that *miR156a* mediated the repression of PpSPL targets by the Dual-Luciferase transient expression assay. The results in this study emphasize the biological functions of *miRNA156a*-SPLs modules in bamboo flowering.

## 5. Conclusions

In this study, a total of 179 miRNAs were identified from *P. pygmaeus* by high-throughput sRNA sequencing, of which 96 were differentially expressed in the shoot buds at different growth stages, which were predicted to target a total of 2099 transcription factors (TFs). Based on previous reports, we identified 23 known miRNAs that are closely related to flowering in plants. A total of 11 of them were significantly differentially expressed between the shoot buds and flower buds in flowering *P. pygmaeus*, which had 124 TF targets corresponding to 132 DEM-TF pairs. In particular, we focused on the identification of the two significantly differentially expressed miRNAs in the age pathway, *miRNA156a* and *miRNA172b*, which are well-known in regulating the vegetative-to-reproductive phase transition in plants. The relative expression level of *miR156a* was significantly higher in the shoot buds than in the flower buds, while *miR172b* displayed the opposite trend. Notably, *miR156a* and *miR172b*, had 36 and 24 TF targets, including 11 SPL and two AP2-like TFs, respectively. It was confirmed that *miR156a* mediated the repression of its *PpSPL* targets by the Dual-Luciferase transient expression assay. The integrated analysis of miRNAs and TF targets at the genome scale in this study reveals that *miR156a*-*PpSPL* modules play a significant role in the floral transition of *P. pygmaeus*. However, their specific mechanisms of action in bamboo flowering are unclear to date, requiring more in-depth research.

## Data Availability

The raw sequencing data are available in Sequence Read Archive (SRA) with accession numbers PRJNA1128838 and PRJNA648794 at the National Center for Biotechnology Information (NCBI).

## Supplementary Materials

The following supporting information can be downloaded at: https://www.mdpi.com/article/10.3390/plants13213033/s1. Figure S1: *Pleioblastus pygmaeus* samples used for small RNA Illumina sequencing; Figure S2: GO analysis of the target genes of differentially expressed miRNAs; Figure S3: KEGG enrichment analysis of the target genes of differentially expressed miRNAs; Table S1: The primers used for gene cloning and the Dual-Luciferase transient expression assay; Table S2: The information of the 120 known miRNAs; Table S3: The information of the 59 novel miRNAs; Table S4: Differentially expressed known miRNAs related to plant flowering in *P. pygmaeus*; Table S5: Differentially expressed known miRNAs related to flower development in *P. pygmaeus*; Supplemental Excel S1: Differentially expressed miRNAs in each comparison pair; Supplemental Excel S2: miRNA-TFs. Refs. [47,48,49,50,51,52,53,54,55,56,57] were cited in the Supplementary Materials.
