# Supplementary material for: Integrated Analysis of microRNAs and Transcription Factor Targets in Floral Transition of Pleioblastus pygmaeus
Source: Plants (Basel). 2024 Oct 30;13(21):3033. doi: 10.3390/plants13213033 (PMC11548222; doi:10.3390/plants13213033)

Figure S1. *Pleioblastus pygmaeus* samples used for small RNA Illumina sequencing

A. Shoot buds of flowering plants (F1); B. Flower buds of flowering plants (F2); C. Shoot buds of non-flowering plants (N1); D. Leaf buds of non-flowering plants (N2). Bar=1 cm.

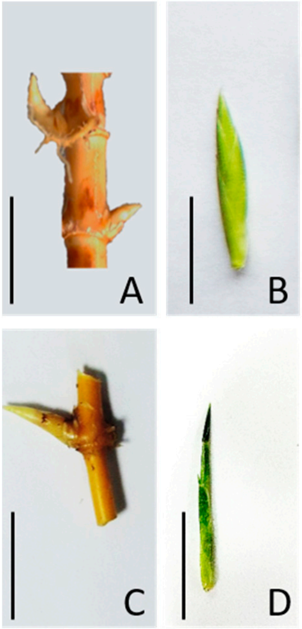

Figure S2. Go analysis of the target genes of differentially expressed miRNAs

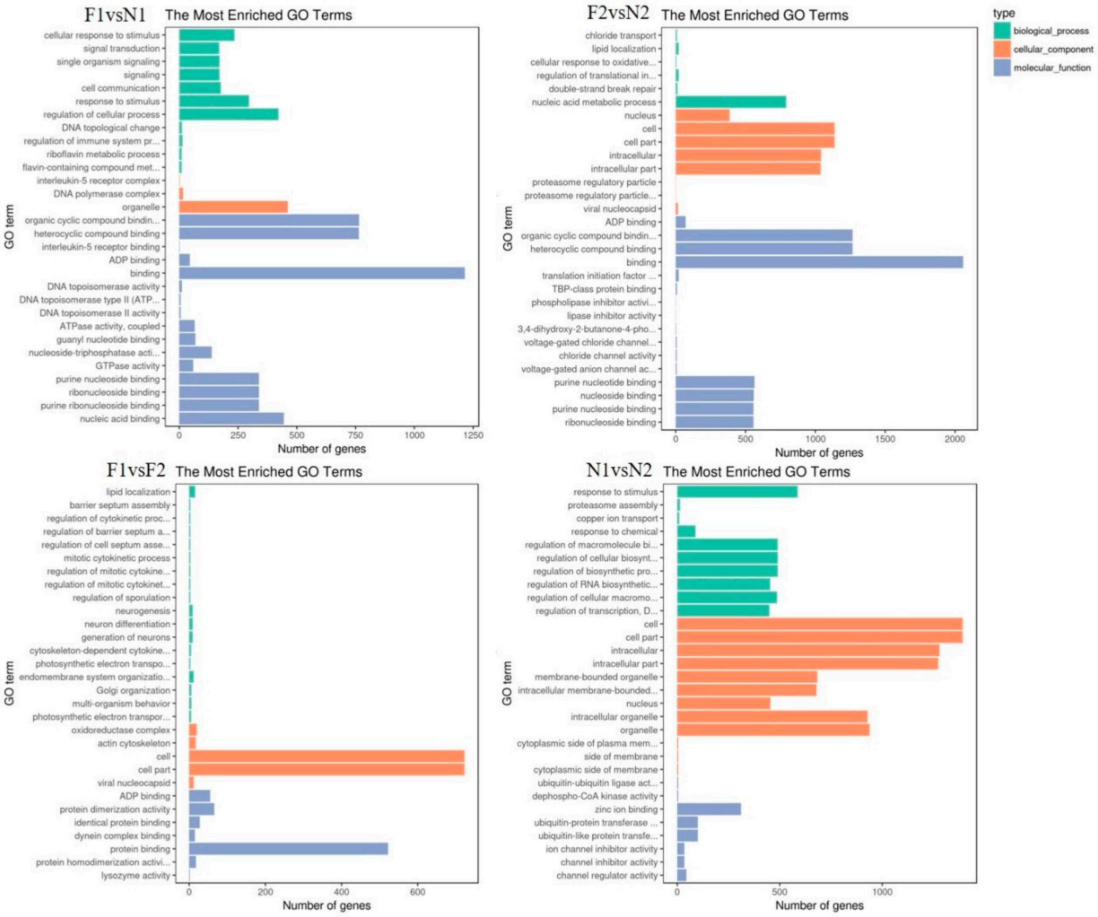

Figure S3. KEGG enrichment analysis of the target genes of differentially expressed miRNAs

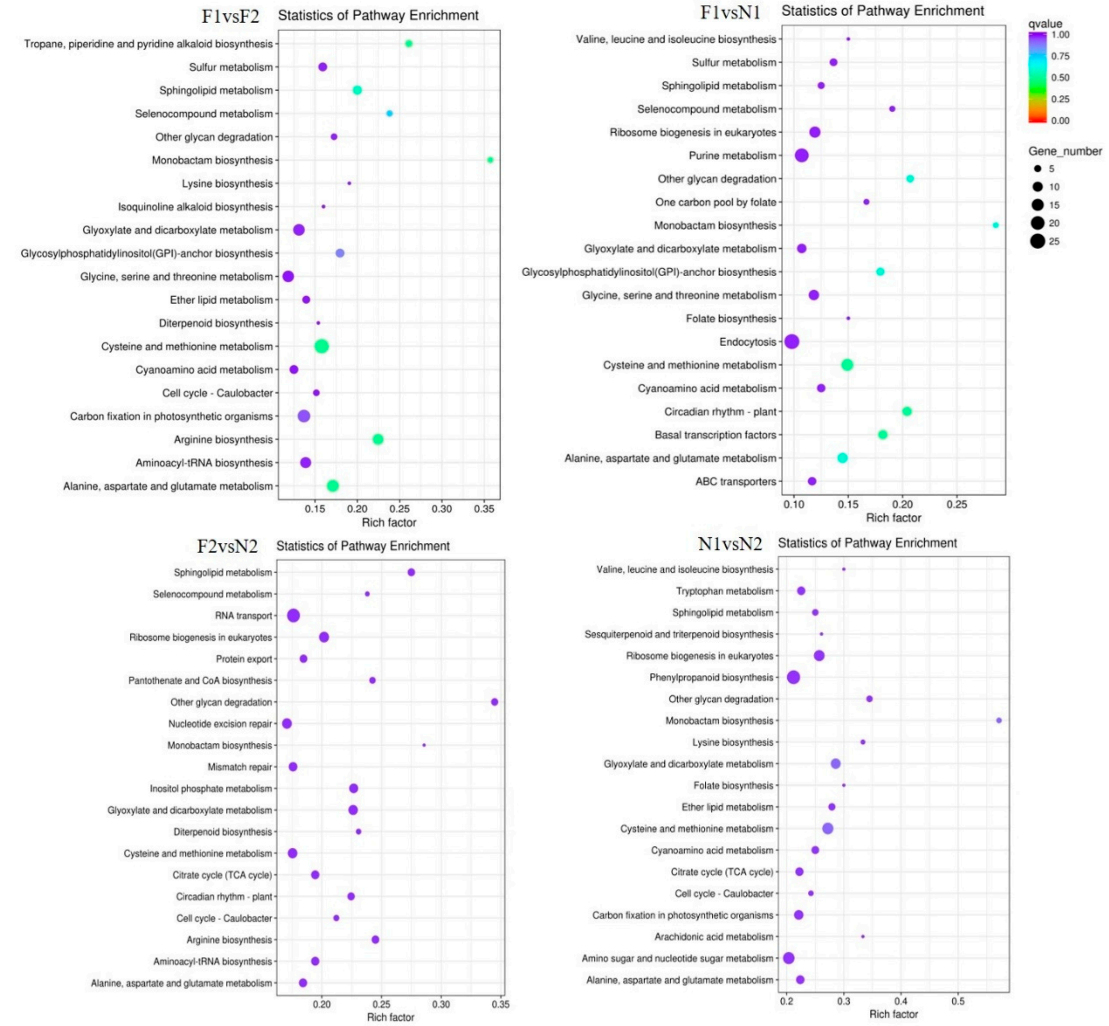

Supplement: Supplementary file 1 [file plants-13-03033-s001.zip › Supplementary Figures.pdf]
