# Supplementary material for: Integrated Analysis of microRNAs and Transcription Factor Targets in Floral Transition of Pleioblastus pygmaeus
Source: Plants (Basel). 2024 Oct 30;13(21):3033. doi: 10.3390/plants13213033 (PMC11548222; doi:10.3390/plants13213033)
Supplement: Supplementary file 1 [file plants-13-03033-s001.zip › Supplementary Tables.pdf]

Table S1 The primers used for gene clone and Dual-Luciferase transient expression assay and and qRT-PCR

Primer	Sequence (5'-3')
Luc-C5	GGAGGAGTTGTGTTTGTGG
35S polyA	TGCTCAACACATGAGCGAAA
LUC-PpSPL12-F	cgcggatccactagtgaattcTTTGTTGGCTCTACATCAACTTACAC
LUC-PpSPL12-R	actggtgatttcagcgaattcGCTTATGACAGTGTTCAGCTGCTC
LUC-PpSPL13-F	cgcggatccactagtgaattcGTCGGCTCTACATCAACTTACACC
LUC-PpSPL13-R	actggtgatttcagcgaattcCGGGACCAGGACGGTGTT
LUC-PpSPL14-F	cgcggatccactagtgaattcGCTATTCAGCCCGGCGAC
LUC-PpSPL14-R	actggtgatttcagcgaattcGTGCAGCGCAGCCTCATG
LUC-PpSPL16-F	cgcggatccactagtgaattcGACCGCATGTCTGGTACTATCCA
LUC-PpSPL16-R	actggtgatttcagcgaattcATGGACCTCGTTCAGCGCA
miR172b-RT	GTCGTATCCAGTGCAGGGTCCGAGGTATTCGCACTGGATACGACATGCAG
miR156a-RT	GTCGTATCCAGTGCAGGGTCCGAGGTATTCGCACTGGATACGACGTGCTC
U6-qR and RT	AATTTGGACCATTTCTCGATTTATGCGTGT
miR156a-qF	CGCGCGTGACAGAAGAGAGT
miR172b-qF	CGCGGGAATCTTGATGATG
U6-qF	GGACATCCGATAAAATTGGAACGATACAG

Table S2 The information of 120 known miRNAs

miRNA family	miRNA name	Mature Sequence (5'-3')	Length (nt)
miR1432	miR1432-5p	AUCAGGAGAGAUGACACCGAC	21
miR156	miR156a	UGACAGAAGAGAGUGAGCAC	20
	miR156c-3p	GCUCACUUCUCUCUCUGUCAGC	22
	miR156f-3p	GCUCACUUCUCUUUCUGUCAGC	22
	miR156j-3p	GCUCGCUCCUCUUUCUGUCAGC	22
	miR156k	UGACAGAAGAGAGAGAGCACA	21
	miR156l-5p	CGACAGAAGAGAGUGAGCAUA	21
miR159	miR159a.2	UUGCAUGCCCCAGGAGCUGCA	21
	miR159a.1	UUUGGAUUGAAGGGAGCUCUG	21
	miR159c	AUUGGAUUGAAGGGAGCUCCA	21
	miR159d	AUUGGAUUGAAGGGAGCUCCG	21
	miR159e	AUUGGAUUGAAGGGAGCUCCU	21
	miR159f	CUUGGAUUGAAGGGAGCUCUA	21
miR160	miR160a-3p	GCGUGCAAGGAGCCAAGCAUG	21
	miR160a-5p	UGCCUGGCUCCCUGUAUGCCA	21
	miR160c-3p	GCGUGCACGGAGCCAAGCAUA	21
	miR160d-3p	GCGUGCGAGGAGCCAAGCAUG	21
	miR160e-5p	UGCCUGGCUCCCUGUAUGCCG	21
	miR160f-5p	UGCCUGGCUCCCUGAAUGCCA	21
miR162	miR162a	UCGAUAAACCUCUGCAUCCAG	21
	miR162b	UCGAUAAGCCUCUGCAUCCAG	21
miR164	miR164a	UGGAGAAGCAGGGCACGUGCA	21
	miR164c	UGGAGAAGCAGGGUACGUGCA	21
	miR164d	UGGAGAAGCAGGGCACGUGCU	21
	miR164e	UGGAGAAGCAGGGCACGUGAG	21
miR166	miR166a-5p	GGAAUGUUGUCUGGUUCAAGG	21
	miR166a-3p	UCGGACCAGGCUUCAUUCCCC	21
	miR166b-5p	GGAAUGUUGUCUGGCUCGGGG	21
	miR166d-5p	GGAAUGUUGUCUGGCUCGAGG	21
	miR166e-3p	UCGAACCAGGCUUCAUUCCCC	21
	miR166g-3p	UCGGACCAGGCUUCAUUCCUC	21
	miR166i-3p	UCGGAUCAGGCUUCAUUCCUC	21
	miR166k-3p	UCGGACCAGGCUUCAAUCCCU	21
	miR166l-5p	GGAUUGUUGUCUGGUUCAAGG	21
	miR166m	UCGGACCAGGCUUCAUUCCCU	21
miR167	miR167a-5p	UGAAGCUGCCAGCAUGAUCUA	21
	miR167c-3p	GGUCAUGCUGCGGCAGCCUCACU	23
	miR167d-3p	GAUCAUGCUGUGCAGUUUCAUC	22

miRNA family	miRNA name	Mature Sequence (5'-3')	Length (nt)
	miR167d-5p	UGAAGCUGCCAGCAUGAUCUG	21
miR168	miR168a-3p	GAUCCCGCCUUGCACCAAGUGAAU	24
	miR168a-5p	UCGCUUGGUGCAGAUCGGGAC	21
	miR168b	AGGCUUGGUGCAGCUCGGGAA	21
miR169	miR169a	CAGCCAAGGAUGACUUGCCGA	21
	miR169b	CAGCCAAGGAUGACUUGCCGG	21
	miR169e	UAGCCAAGGAUGACUUGCCGG	21
	miR169f.1	UAGCCAAGGAUGACUUGCCUA	21
	miR169h	UAGCCAAGGAUGACUUGCCUG	21
	miR169r-5p	UAGCCAAGGAUGAUUUGCCUG	21
miR171	miR171a	UGAUUGAGCCGCGCCAAUAUC	21
	miR171b	UGAUUGAGCCGUGCCAAUAUC	21
	miR171c-5p	GGAUAUUGGUGCGGUUCAAUC	21
	miR171e-5p	UGUUGGCUCGGCUCACUCAGA	21
	miR171f-5p	UGUUGGCAUGGUUCAAUCAAA	21
	miR171i-3p	GGAUUGAGCCGCGUCAAUAUC	21
miR172	miR172a	AGAAUCUUGAUGAUGCUGCAU	21
	miR172b	GGAAUCUUGAUGAUGCUGCAU	21
	miR172c	UGAAUCUUGAUGAUGCUGCAC	21
	miR172d-5p	GCAGCACCAUCAAGAUUCAC	20
miR1863	miR1863a	AGCUCUGAUACCAUGUUAGAUUAG	24
	miR1863b	AGCUCUGAUACCAUGUUAACUGUU	24
miR1871	miR1871	AUGGCUCUGAUAUCAUGUUGGUUU	24
miR2118	miR2118a	UUCUCGAUGCCUCCCAUUCCUA	22
	miR2118b	UUCCCGAUGCCUCCCAUUCCUA	22
	miR2118d	UUCCUGAUGCCUCCCAUGCCUA	22
	miR2118e	UUCCCAAUGCCUCCCAUGCCUA	22
	miR2118f	UUCCUGAUGCCUCCCAUUCCUA	22
	miR2118p	UUCCCGAUGCCUCCCAUGCCUA	22
miR2120	miR2120	AAAGAUCUUUAGUCCCGGUUGUUC	24
miR2275	miR2275a	UUUGGUUUCCUCCAAUAUCUCA	22
	miR2275c	AGAAUUGGAGGAAAACAAACUGA	23
	miR2275d	CUUGUUUUUCUCCAAUAUCUCA	22
miR319	miR319a-3p.2-3p	UUGGACUGAAGGGUGCUCCC	20
miR393	miR393a	UCCAAAGGGAUCGCAUUGAUC	21
	miR393b-3p	UCAGUGCAAUCCCUUUGGAAU	21

miRNA family	miRNA name	Mature Sequence (5'-3')	Length (nt)
miR394	miR394	UUGGCAUUCUGUCCACCUCC	20
miR395	miR395b	GUGAAGUGUUUGGGGGAACUC	21
miR396	miR396a-5p	UUCCACAGCUUUCUUGAACUG	21
	miR396a-3p	GUUCAAUAAAGCUGUGGGAA	20
	miR396c-5p	UUCCACAGCUUUCUUGAACUU	21
	miR396c-3p	GGUCAAGAAAGCUGUGGGAAG	21
	miR396e-5p	UCCACAGGCUUUCUUGAACUG	21
	miR396e-3p	AUGGUUCAAGAAAGCCCAUGGAAA	24
	miR396f-5p	UCUCCACAGGCUUUCUUGAACU	22
	miR396f-3p	AUAGUUCAAGAAAGUCCUUGGAAA	24
	miR396g	UCCACAGGCUUUCUUGAACGG	21
miR397	miR397a	UCAUUGAGUGCAGCGUUGAUG	21
	miR397b	UUAUUGAGUGCAGCGUUGAUG	21
miR398	miR398b	UGUGUUCUCAGGUCGCCCCUG	21
miR399	miR399a	UGCCAAAGGAGAAUUGCCCUG	21
	miR399d	UGCCAAAGGAGAGUUGCCCUG	21
	miR399e	UGCCAAAGGAGAUUUGCCCAG	21
	miR399h	UGCCAAAGGAGACUUGCCCAG	21
	miR399i	UGCCAAAGGAGAGCUGCCCUG	21
	miR399j	UGCCAAAGGAGAGUUGCCCUA	21
miR408	miR408-5p	CAGGGAUGAGGCAGAGCAUGG	21
	miR408-3p	CUGCACUGCCUCUUCCCUGGC	21
miR444	miR444a-3p.2	UGCAGUUGCUGCCUCAAGCUU	21
	miR444a-3p.1	UUGCUGCCUCAAGCUUGCUGC	21
	miR444b.2	UGCAGUUGUUGUCUCAAGCUU	21
	miR444b.1	UGUUGUCUCAAGCUUGCUGCC	21
	miR444d.3	UUGUGGCUUUCUUGCAAGUUG	21
	miR444f	UGCAGUUGUUGCCUCAAGCUU	21
miR5072	miR5072	CGAUUCCCCAGCGGAGUCGCCA	22
miR5073	miR5073	GUUUGGUGAAUCGGAAACUAUUU	23
miR5076	miR5076	GAAAUGGGAGCAGAGCAGGUUU	22
miR5082	miR5082	UGCGAUGAUGGCCGCGCGGGUUCA	24
miR5083	miR5083	AGACUACAAUUAUCUGAUCA	20
miR5179	miR5179	UUUUGCUCAAGACCGCGCAAC	21

miRNA family	miRNA name	Mature Sequence (5'-3')	Length (nt)
miR528	miR528-3p	CCUGUGCUUGCCUCUUCCAUU	21
	miR528-5p	UGGAAGGGGCAUGCAGAGGAG	21
miR529	miR529a	CUGUACCCUCUCUCUUCUUC	20
	miR529b	AGAAGAGAGAGAGUACAGCUU	21
miR530	miR530-5p	UGCAUUUGCACCUGCACCUA	20
miR535	miR535-5p	UGACAACGAGAGAGAGCACGC	21
miR5523	miR5523	UGAGGAGGAACAUAUUUACUAG	22
miR5538	miR5538	ACUGAACUCAAUCACUUGCUGC	22
miR5539	miR5539a	AAGAAAACGGAUGCGCGUGCUA	22
miR6246	miR6246	UUGGGGAUUUCCUGCCGGAGGAA	23
miR6253	miR6253	GAGGAAAGUGGGCAGUUGGGUU	22
miR827	miR827	UUAGAUGACCAUCAGCAAACA	21

Table S3. The information of 59 novel miRNAs

miRNA name	Mature Sequence (5'-3')	Length (nt)
novel_1	AUCGCGAUCUUGACAACCUUGACU	24
novel_4	UUUGGAUUGAAGGGAGCUCUG	21
novel_7	AUCUCAGCUGUUGAUUCCAUGAUC	24
novel_10	UCGACGACGCCGUUCUGCUGC	21
novel_11	GAUCAGAUCGUGUAUAUGUAUAUG	24
novel_15	AGCUCUGAUACCAUGUUAGGAUUG	24
novel_17	GUGAUUUCGGUACUUGUAAUACA	23
novel_18	AGGUUUACUAUUCGUUGGAGCAGU	24
novel_19	UGCUCACUGCUCUGUCUGUCAUC	23
novel_20	UUCAGUUUCCUCUAAUAUCUCA	22
novel_21	UGGGUCCGUGAACUUGAUAAC	21
novel_22	UGUUGGCUCGUCUCACUCAGA	21
novel_23	UUGUAAACUGCACGACUCCUU	21
novel_24	GAGUGUCUUGUGCAGUGAACCGUG	24
novel_25	UGAAAGUGGUAACUUGUAGAUGGU	24
novel_26	UGGGCUGGUUUCAUGAGUGGU	21
novel_27	AGGCUCGACGGAUUGGAACACGCC	24
novel_28	UGUGUUCUCAGGUCGCCCCUG	21
novel_29	AGGAAAAAAAUUCUAUGGGACGGG	24
novel_30	UGCACUGCCUCUUCCCUGGG	20
novel_31	CAUGUGCCCGUCUUCUCCACC	21
novel_33	UAUUUGGGUGAUUUUGGUAUU	21
novel_35	UCAGAUCUCAUCCAAUGACUCAUG	24
novel_36	UGAUCUUAAUCCGUUGUAUUGUCA	24
novel_37	AGAUAUUGGCGUGCCUCAGUC	21
novel_42	UUGGCGCUCUAAUUCAUUUUCUU	23
novel_43	UUCCUUGACUCGUUGGUAG	19
novel_44	UGGCUAGUGGCGCUUAACCUCGGG	24
novel_45	CAUGUGCCCUUCUUCUCCACC	21
novel_46	AAAGAUAGUAUAUGCACCCACAUG	24
novel_47	AGCUGUACCUGAGAGUCUGGACGG	24
novel_48	CUUGUUCUUCUCCAAUAUCUCA	22
novel_50	UUGGACGUGUUCUUGAUA	18
novel_51	AGUGUCUGCUGGUAGAACAUCAGA	24
novel_52	ACGGGUCUAGGUAAUAAUUUCGCA	24
novel_53	UUUCAAAUUCUGACCCCUUUGAUC	24
novel_55	ACGGUUGGGCGACUGGAUCCUAUC	24
novel_56	UUACCGGCCGGGAUUUUGAUCC	22
novel_57	AAAACACAUUUGCCGACUUUUAGA	24
novel_58	CAAGGGCUGAAGCGUGUAAGGAGA	24
novel_59	UUUGACGGGGAACCCAGCUUU	21
novel_60	UCGAUAAACCUCUGCAUCCGG	21

miRNA name	Mature Sequence (5'-3')	Length (nt)
novel_64	UCGCAGGAGAGAUGACGCCCA	21
novel_65	UGUGUUCUCAAUUCGCCCCUG	21
novel_66	UGGACUGAAGGGUGCUCCCUC	21
novel_67	UAGCCAAGGAUGAUUUGCCGG	21
novel_68	AGGCACGGCACUGUAGGCCCGACC	24
novel_69	AAUAGGUGUCAGACUAUUGACGUG	24
novel_70	AUUUUAGGAUCUACCACUCGAUGU	24
novel_71	UUUUGAGGCAUUAGUAACAUA	21
novel_72	AUCGCCCUCCUGGUCGCGGCUGGG	24
novel_73	ACUGGAUCUUUUUAAGUAGUAUAUA	25
novel_74	AUACUCGGCCGUAUACAAGUAACG	24
novel_75	AUCGAUCUCUUCUGUUCAACAAAA	24
novel_76	AAUUUGCCGGUUGCUGGUUCUACC	24
novel_77	AAAACCGUCUGUGAUGACCUCAUG	24
novel_78	GUGCUCCCUCCCGUUGUCACUCCU	24
novel_79	UGACUGUACCUAUGUGGUAGC	21
novel_81	AGGCUUACUAUUCGUUGGAGCAGU	24

Table S4 Differentially expressed known miRNAs related to plant flowering in *P. pygmaeus*

Family	Members	Target genes	Target gene annotation	Biological function of miRNAs
miR156*3p*	*miR156a* *miR156f-3p* *miR156j-3p*	Cluster-21960.0 Cluster-10432.20508 Cluster-10432.2297 Cluster-15920.0 Cluster-4416.0 Cluster-10432.6783 Cluster-10432.12439 Cluster-7251.0 Cluster-10432.20509 Cluster-10432.28485 Cluster-10432.22213	SQUAMOSA promoter-binding protein-like (SPL) transcription factor	In Arabidopsis, overexpression of *miR156* significantly prolongs juvenile phase, leading to delayed flowering. It down-regulates the expression of 11 SPL transcription factors by transcript cleavage [8,15].
miR159	*miR159a.1* *miR159c* *miR159d* *miR159e* *miR159f*	Cluster-9249.0 Cluster-10432.4081 Cluster-10432.4086 Cluster-10432.19759	GAMYB and MYB transcription factor	Overexpression of *miR159a* showed delayed flowering, and the relative expression levels of MYB33 and LFY showed a decreasing trend in transgenic Arabidopsis [19].
miR168	*miR168a-3p* *miR168a-5p*	Cluster-14895.0 Cluster-10432.17509 Cluster-10432.14756 Cluster-10432.25992	Protein argonaute (AGO)	Inhibiting *miR168* can shorten flowering time and increase yield in rice [47].
miR169	*miR169b*	Cluster-14873.0 Cluster-17965.0 Cluster-1276.0 Cluster-10432.27496 Cluster-11129.0	Nuclear transcription factor Y subunit A(NF-YA)	The up-regulation of *miR169* by abiotic stress reduces the expression of AtNF-YA transcription factor, result in early flowering in Arabidopsis [20].
miR171	*miR171a* *miR171b*	Cluster-10432.10918	Scarecrow-like protein (SCL) transcription factor	*MiR171* regulates late flowering and leads to reduced expression of *HvSCL* genes in barley [48].

Family	Members	Target genes	Target gene annotation	Biological function of miRNAs
	miR171e-5p *miR171i-3p*	Cluster-10432.19167 Cluster-10432.20480 Cluster-10432.23772		
miR172	*miR172b*	Cluster-11953.0 Cluster-10432.7960	APETALA2-like protein (AP2) transcription factor	Overexpression of *miR172* significantly promotes flowering in Arabidopsis. And overexpression of its target gene, *AP2*-like gene, results in delayed flowering[16, 18].
miR393	*miR393b-3p*	Cluster-10432.9477 Cluster-10432.15882	Transport inhibitor response 1-like protein (TIR), AFB1 (Auxin Signaling F-box Protein)	*MiR393* regulates TIR1 and AFB1-3. Overexpression of a *miR393*-resistant form of TIR1 (*mTIR1*) led to delayed flowering in Arabidopsis [49].
miR394	*miR394*	Cluster-10432.14023 Cluster-10432.19588	F-box protein	*Mir394* mutants exhibit early flowering, lower expression of floral repressor FLC and higher expression of floral integrators FT and SOC1. *miR394* up-regulates the expression of a F-Box protein [50].
miR397	*miR397a* *miR397b*	Cluster-10432.6056	Laccase (LAC)	*MiR397b* delays flowering by targeting *CKB3*, in turn modulating the circadian period of *CCA1* in Arabidopsis [51].
miR399	*miR399d*	Cluster-1496.0 Cluster-13663.0	Phosphate (PHO), NAC transcription factor	Both the transgenic Arabidopsis overexpressing miR399 and the mutant Arabidopsis of its target gene, *PHO2*, exhibit early flowering phenotype [52].
miR528	*miR528-3p* *miR528-5p*	Cluster-16512.0 Cluster-10432.2863 Cluster-10432.25552 Cluster-10432.2425	RFI, WRKY, NAC, AP2/ERF, bHLH transcription factor	*MiR528* promotes rice flowering by inhibiting the expression of *OsRFI2* under long day condition [53].

Note: The 11 miRNAs marked in red are significantly differentially expressed in the different tissues of *P. pygmaeus*.

Table S5. Differentially expressed known miRNAs related to flower development in *P. pygmaeus*

Family	Members	Target genes	Target gene annotation	Biological function of miRNAs
miR164	*miR164c*	Cluster-18122.1<br>Cluster-10432.14330<br>Cluster-10432.27018<br>Cluster-10432.11791<br>Cluster-10432.24191	NAC transcription factor	MiR164 enlarges the sepal/sepal boundary, by degrading CUC1 and CUC2 mRNAs in Arabidopsis [54].
miR167	*miR167d-5p*	Cluster-10432.14968	Auxin response factor (ARF)	MiR167 negatively regulates ARF6 and ARF8. Arabidopsis mARF6 or mARF8 plants with mutated miR167 target sites have defective anther dehiscence and ovule development in Arabidopsis [55].
miR2118	*miR2118d*<br>*miR2118e*<br>*miR2118p*	Cluster-10432.4290<br>Cluster-10432.17500	MYB transcription factor	Mir2118 rice mutants showed both m ale and female photoperiodic sterility[56].
miR396	*miR396a-5p*	Cluster-5368.0<br>Cluster-7949.0<br>Cluster-20231.0<br>Cluster-7703.0<br>Cluster-23610.0<br>Cluster-3685.0<br>Cluster-10432.5988<br>Cluster-10432.5987<br>Cluster-6403.0<br>Cluster-21332.1<br>Cluster-842.0<br>Cluster-21332.0<br>Cluster-10432.25921<br>Cluster-10432.11270<br>Cluster-351.0	Growth-regulating factor (GRF)	The miR396–GRF/GIF module is required for cotyledon and floral organ separation, floral organ growth and reproductive development [57].
